# Central modulation in cluster headache patients treated with occipital nerve stimulation: an FDG-PET study

**DOI:** 10.1186/1471-2377-11-25

**Published:** 2011-02-24

**Authors:** Delphine Magis, Marie-Aurélie Bruno, Arnaud Fumal, Pierre-Yves Gérardy, Roland Hustinx, Steven Laureys, Jean Schoenen

**Affiliations:** 1 Headache Research Unit and Neurology Department, University of Liège, CHR Citadelle, Boulevard du 12ème de Ligne 1, 4000 Liège, Belgium; 2 Cyclotron Research Centre and Neurology Department. Coma Science Group, University of Liège, Belgium; 3 Headache Research Unit and Neurology Department, University of Liège, CHR Citadelle, Boulevard du 12ème de Ligne 1, 4000 Liège, Belgium; 4 Headache Research Unit and Neurology Department, University of Liège, CHR Citadelle, Boulevard du 12ème de Ligne 1, 4000 Liège, Belgium; 5 Nuclear Medicine Department, University of Liège, CHU Sart-Tilman, 4000 Liège, Belgium; 6 Cyclotron Research Centre and Neurology Department. Coma Science Group, University of Liège, Belgium; 7 Headache Research Unit and Neurology Department, University of Liège, CHR Citadelle, Boulevard du 12ème de Ligne 1, 4000 Liège and GIGA-Neurosciences, University of Liège, Belgium

## Abstract

**Background:**

Occipital nerve stimulation (ONS) has raised new hope for drug-resistant chronic cluster headache (drCCH), a devastating condition. However its mode of action remains elusive. Since the long delay to meaningful effect suggests that ONS induces slow neuromodulation, we have searched for changes in central pain-control areas using metabolic neuroimaging.

**Methods:**

Ten drCCH patients underwent an ^18^FDG-PET scan after ONS, at delays varying between 0 and 30 months. All were scanned with ongoing ONS (ON) and with the stimulator switched OFF.

**Results:**

After 6-30 months of ONS, 3 patients were pain free and 4 had a ≥ 90% reduction of attack frequency (responders). In all patients compared to controls, several areas of the pain matrix showed hypermetabolism: ipsilateral hypothalamus, midbrain and ipsilateral lower pons. All normalized after ONS, except for the hypothalamus. Switching the stimulator ON or OFF had little influence on brain glucose metabolism. The perigenual anterior cingulate cortex (PACC) was hyperactive in ONS responders compared to non-responders.

**Conclusions:**

Metabolic normalization in the pain neuromatrix and lack of short-term changes induced by the stimulation might support the hypothesis that ONS acts in drCCH through slow neuromodulatory processes. Selective activation in responders of PACC, a pivotal structure in the endogenous opioid system, suggests that ONS could restore balance within dysfunctioning pain control centres. That ONS is nothing but a symptomatic treatment might be illustrated by the persistent hypothalamic hypermetabolism, which could explain why autonomic attacks may persist despite pain relief and why cluster attacks recur shortly after stimulator arrest. PET studies on larger samples are warranted to confirm these first results.

## Background

Cluster headache (CH) is one of the most painful primary headaches and is characterized by attacks of severe unilateral periorbital pain associated with ipsilateral autonomic features [1]. About 10% of patients have, or develop over time, a chronic form (CCH) [2] characterised by recurrent attacks for at least 1 year without remissions or with remissions of less than 1 month [1]. About 1% of CCH patients become drug-resistant (drCCH) to most prophylactic drug treatments and fulfil published criteria for intractable headaches [3].

CH is the most prevalent member of the so-called trigeminal autonomic cephalalgias (TACs), which include paroxysmal hemicrania, SUNCT (Short-lasting Unilateral Neuralgiform headache with Conjunctival injection and Tearing) and probably hemicrania continua [4]. Neuroimaging studies have provided new insight into the pathophysiology of these disorders. Besides non-specific changes in activity of brain areas belonging to the pain matrix like the anterior cingular cortex (ACC), insula(e), and thalamus, TACs are associated with ictal activation of ipsilateral posterior hypothalamus (CH, SUNCT) or dorsal pons (hemicrania continua) or contralateral posterior hypothalamus (paroxysmal hemicrania) which may be more specific and disease-related [5].

As a consequence, deep brain stimulation (DBS) targeting the posterior hypothalamus was proposed for drCCH and was found to be more effective than any previously used invasive therapy [6,7]. However hypothalamic DBS is not a riskless procedure [7] and less invasive methods were thus explored. Among them, occipital nerve stimulation (ONS) had comparable efficacy to hypothalamic DBS, except for slower onset of action [8,9].

The mechanisms by which ONS improves drCCH remain unclear. In a study of ONS in drCCH we found no significant change in pain thresholds, which argues against a diffuse analgesic effect [8]. It was speculated that ONS might exert its action by decreasing excitability of second order nociceptors in trigeminal nucleus caudalis on which converge cervical, somatic trigeminal and visceral trigeminovascular afferents [10,11]. Yet, the nociception-specific blink reflex, mediated by spinal trigeminal nucleus interneurons, was increased rather than decreased in our study of ONS in drCCH [8] and it remained unchanged in healthy subjects after short low frequency transcutaneous stimulation of the greater occipital nerve [12]. A more likely explanation for the therapeutic effect of ONS in headache including drCCH is the induction of slow neuromodulatory changes in brain regions belonging to the pain matrix or in centres more specifically involved in CCH pathophysiology. Hence, chronic migraine patients treated with ONS [13] show significant blood flow increases on H2^15^O-PET in dorsal rostral pons, anterior cingulate cortex and cuneus, directly correlated to pain scores, and in left pulvinar, inversely correlated to such scores. Dorsal pons activation persisted after ONS, supporting the role of this structure in migraine pathophysiology [13].

So far, functional imaging studies have not been performed in ONS-treated drCCH patients. We performed such a study focusing on the pain matrix, but also on hypothalamus and brainstem that seem more specifically involved in the pathophysiology of TACs. We enrolled patients from the published cohort [8] and newly implanted ones. We used 18-fluorodeoxyglucose-positron emission tomography (18-FDG-PET) in order to detect long term activity modulation.

## Methods

### Subjects

We studied 10 patients with drCCH (9 males and 1 female, mean age at implantation 44.2 ± 9.9 years SD). Inclusion criteria were: CCH for at least 2 years, daily attacks by history, side-locked attacks from the beginning, resistance to drug treatment according to expert consensus guidelines [3] and absence of associated disabling organic or psychiatric disorder. Five patients had left-sided, 5 right-sided attacks. At the time of the study, all patients were taking one or several of the following preventive drugs: verapamil (n = 9), lithium carbonate (n = 6), methylprednisolone (n = 2), methysergide (n = 2), melatonine (n = 1), gabapentine (n = 1). None took analgesics, in particular opioids.

Patients were recruited in two phases (1^st ^and 2^nd ^group), with written informed consent. Approval of the local Ethics Committee for ONS in drCCH was first obtained for 5 patients (EUDRACT-2004-004551-19). Because of the favourable results in ONS-treated patients after a 16 months follow-up, we requested Ethics Committee approval for a protocol amendment allowing us to recruit 6 supplementary patients who were implanted and agreed to undergo PET before and after surgery. At the same time, patients of the 1^st ^group were also asked to participate in the PET study and 4 of them accepted (the last patient of group 1 had been explanted [8]).

### Surgical and stimulation procedure

Surgical procedure and stimulation protocols have been described previously [8]. We used unilateral subcutaneous implantation of paddle style stimulating leads with 4 electrode plots (Medtronic 3587A Resume II^®^; Medtronic Inc., Minneapolis, USA) via a retromastoid C1-2-3 approach [14] and Medtronic Itrel III^® ^or Synergy^® ^stimulators.

Stimulation protocols were adapted using a programming matrix [8] such as to induce paraesthesias in the largest possible occipital territory. The clinical evolution of patients was monitored with cluster headache paper diaries.

### 18-FDG-PET study design

#### Study groups

In the 1^st ^group, patients (n = 4), underwent a PET session after 24 to 30 months of ONS. All patients but one belonging to the 2^nd ^group (n = 6) were scanned before implantation (baseline). In both groups, for each session after implantation, patients were scanned with the stimulator switched on (ON) and off for 3 days (OFF). This period was arbitrarily fixed in line with our previous observation of headache recurrence within 2 days on average after switching off the stimulator [8]. They underwent 2 more pairs of scans after 1 and 6 months of ONS. None of the patients had a cluster attack during PET, nor within the 12 hours preceding or following the procedure.

Data collected in patients were compared to a pool of 39 drug-free healthy volunteers (HV) (18 males, 21 females, mean age 45 ± 16 years SD) without headache history.

#### Data acquisition

PET data were obtained on a Siemens CTI 951 16/32^® ^scanner (Siemens, Erlangen). Data were corrected for attenuation and background activity. Resting cerebral metabolism was studied after intravenous injection of 5-10 mCi (185-370 MBq) [18F]fluorodeoxyglucose (FDG). Subjects were scanned in a dark room, with minimal environmental noise.

#### Statistical analysis (Cyclotron Research Centre, Liège)

Because we selected CH patients with side-locked unilateral attacks and as there was no side shift during the observational period, we flipped the PET scans of the patients with right-sided symptoms in the axial plane so that we could analyze all subjects together (all "left-sided"). We used a binary classification of patients responding or not to ONS with an arbitrary, but clinically relevant, cut-off point for responders set at 50% decrease in attack frequency. Given the data from previous functional imaging studies, we conducted our analysis with an a priori hypothesis towards regions known to be involved in CH and other TACs [5], areas which are modulated by ONS in chronic migraine [13] and areas belonging to the pain matrix.

Data were analysed using statistical parametric mapping (SPM8 version; Wellcome Department of Cognitive Neurology Institute of Neurology, London, UK; http://www.fil.ion.ucl.ac.uk/spm) implemented in MATLAB (version 7.1, MathworksInc., Sherborn, MA). Images were spatially normalized into a standard stereotactic space using a symmetrical MNI (Montreal Neurological Institute) PET template [15] and smoothed using a 14 mm full-width-half-maximum (FWHM) isotropic kernel [16]. T-test was used with a significance level set at p < 0.001 uncorrected (p < 0.05 FDR).

The first design matrix included the scans of the 39 HV, of the 4 patients of the 1^st ^group performed 24 to 30 months post-ONS and of the 6 patients of the 2^nd ^group, scanned before implantation (baseline), 1 month and 6 months post-ONS. Our first analysis identified brain regions with a significant hyper- or hypometabolism in drCCH patients as compared to HV independent on time of scanning or ONS stimulation. Then, we looked for brain regions showing short term ONS-induced (ON versus OFF) increase or decrease in metabolism independent on delay since ONS implantation, in the early post-ONS phase (scans obtained after 1 month) and in the late post-ONS phase (scans obtained after 6 and 24-30 months). Finally, we compared metabolic activity measured during baseline, early phase (1 month) and late phase (≥ 6 months) post-ONS (independent of ONS stimulator settings) searching for progressive increases and decreases in metabolism over time.

In a second design matrix we searched for differences between the subgroups of 7 responders and 3 non-responders. Here, we included only the PET data obtained in the late phase (≥ 6 months; both with stimulator ON and OFF) and the HV scans, searching for regions with metabolic differences between the two groups (i.e., increased metabolism - as compared to HV - present in responders but not in non-responders).

For all analyses, the resulting set of voxel values for each contrast, constituting an SPM of the t-statistics (SPM{t}), was transformed to the unit normal distribution (SPM{Z}) and thresholded at p = 0.001. All group results were thresholded at false discovery-corrected p < 0.05, corrected for the whole brain volume. For differences between responder and non-responder subgroups, results were corrected for multiple comparisons within the regions of interest identified during the previous whole group analyses by employing a small volume correction (10 mm radius sphere).

## Results

### Clinical outcome

Clinical data of patients and changes in their attack frequency after various durations of ONS are summarized in table 1. All patients but one in the 1^st ^group (N = 4, 24-30 months follow-up) improved after ONS: one patient was pain free; 2 patients had a 90% and 93% reduction in attack frequency; the "non-responder" patient had a 25% improvement. In the 2^nd ^group (N = 6), at 6 months follow-up, 3 patients were pain free and one was improved by 90%; these 4 patients already reported significant improvement after 1 month of ONS. The 5^th ^patient only had a 33% reduction in attack frequency while in the 6^th ^patient, attack frequency was slightly increased. According to the 50% cut-off criterion, 7 patients were thus considered responders and 3 non-responders to ONS for the binary analysis. During the 3-day period of ONS interruption, only one responder had recurrence of attacks. In the 2^nd ^group, all patients kept the same preventive drug treatment during the follow-up scans except for one responder (8) who was able stop all medications after 6 months.

**Table 1:** Patients characteristics and clinical outcome

Patients	Age	Cluster & ONS side	Average number of attacks/ last 4 weeks			% reduction in attack frequency		ONS responder
			**BEFORE** **ONS**	**24-30 mths** **ONS**		**24-30 mths** **ONS**		
				
***Group 1***								
1	48	R	131.6	98.0		26		N
2	46	L	107.6	7.6		93		**Y**
3	32	R	32.4	3.2		90		**Y**
4	53	L	28.0	0.0		100		**Y**
			**BEFORE** **ONS**	**1 month** **ONS**	**6 months** **ONS**	**1 month** **ONS**	**6 months** **ONS**	
				
***Group 2***								
5	31	R	28.0	7.2	0.0	74	100	**Y**
6	60	R	28.0	7.2	2.8	74	90	**Y**
7	47	L	112.0	92.4	116.4	17.5	-4	N
8	50	R	42.0	4.4	0.0	80	100	**Y**
9	31	L	56.0	2.0	0.0	97	100	**Y**
10	44	L	16.4	13.2	11.2	22	33	N

ONS: occipital nerve stimulation, R: right, L: left, Y: yes, N: no.

### PET results

The main areas of peak voxels where a metabolic change was found for the various comparisons are shown in table 2.

**Table 2:** Main statistical results and localization of peak voxels where cerebral metabolism was activated (>) or deactivated (<)

Analysis	Brain region	Talairach coordinates			Z score of peak	p FDR corrected
		x	y	z		
drCCH > HV	ACC	12	40	-4	5	< 0.001
	Perigenual ACC	-8	28	-8	5.29	< 0.001
	Midcingulate	12	20	30	4.07	0.003
	Left visual cortex	-10	-98	-8	3.43	0.010
	Left pulvinar	-16	-36	8	4.49	0.001
	Left hypothalamus	-2	-12	-16	2.71	0.013
	Cerebellum	-16	-36	46	4.28	0.002
	Midbrain	2	-34	-4	3.53	0.008
	Left lower pons	-8	-32	-46	4.13	0.003

drCCH < HV	R/L Sensorimotor	-58	-34	-18	4.43	0.012
		58	-18	-26	4.42	0.012
		-4	-22	70	4.26	0.012
		-25	-22	66	3.73	0.015
		-6	-24	68	3.37	0.025
	Right prefrontal	42	26	24	3.76	0.015

Base > ONS	ACC	-8	28	-8	4.66	0.003
	Midcingulate	12	20	30	3.75	0.013
	Left visual cortex	-10	-98	-10	3.25	0.031
	Left pulvinar	-18	-38	6	3.8	0.012
	Cerebellum	2	-42	-16	3.67	0.015
	Midbrain	2	-40	-12	3.63	0.016
	Left lower pons	-8	-30	-44	4.40	0.024

Base < ONS	R/L Sensorimotor	-58	-34	-18	4.59	0.013
		58	-18	-26	4.26	0.037
		42	26	24	3.75	0.026
		-4	-22	70	3.78	0.029

Resp > non resp	Perigenual ACC	-8	28	-8	4.01	0.002*

Coordinates are in the standardized stereotactic Montreal Neurological Institute space (mm). FDR = False discovery rate corrected. drCCH: drug-resistant chronic cluster headache; HV: healthy volunteers; ACC: anterior cingulate cortex, R: right, L: left. * FDR corrected in region of interest identified in whole group analysis

**Figure 1:**
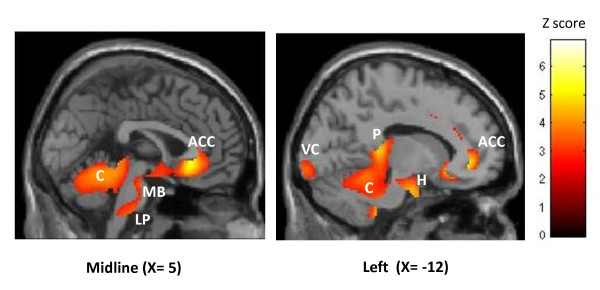
**Hypermetabolic areas in drCCH patients (all conditions: baseline - 1 month, 6 months, 24/30 months) compared with HV (p < 0.05 FDR corrected)**. Results are displayed on 2 sagittal sections of a normalized MRI template (through midline and left hemisphere). ACC: anterior cingulate cortex, C: cerebellum, MB: midbrain, LP: lower pons, VC: visual cortex, P: pulvinar, H: hypothalamus.

We first pooled all scans performed in drCCH patients and compared them with those of the HV. In comparison to HV, a significant hypermetabolism was found in anterior cingulate cortex (ACC), left hypothalamus, left pulvinar, left visual cortex, cerebellum and brain stem (left lower pons and midbrain) (figure 1). By contrast, a significant hypometabolism appeared in both sensori-motor areas.

There was no significant difference between scans performed with stimulator ON or OFF, regardless of ONS duration (1, 6 or 24-30 months). For scans obtained in the late phase (≥ 6 months) we observed a hypermetabolism in the left frontal lobe (BA 10, uncorrected p = 0.03, x = -12, y = 46, z = 4) and the left lower brainstem (uncorrected p = 0.014, x = -6, y = -30, z = -36) when the stimulator was turned ON, but these results, reported for the sake of completeness, did not survive correction for multiple comparisons.

**Figure 2:**
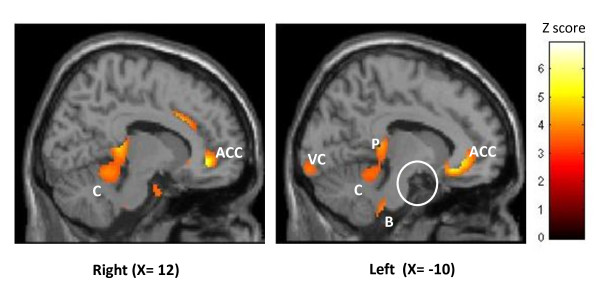
**Areas progressively deactivated by ONS over time (p < 0.05 FDR corrected)**. Results are displayed on 2 sagittal sections of a normalized MRI template (right and left hemisphere). ACC: anterior cingulate cortex, C: cerebellum, B: brainstem, VC: visual cortex, P: pulvinar. White circle highlights hypothalamic area, which is not modified by the stimulation.

Over time, ONS changed glucose uptake in several brain areas (independent of the stimulator settings ON or OFF). The anterior cingulate, mid cingulate, left pulvinar, midbrain, lower pons, visual cortex and cerebellum had decreased metabolism over time, i.e. they became less hypermetabolic when comparing baseline to the early phase (1 month) or the late phase (≥ 6 months). By contrast, metabolism increased over time in sensorimotor cortices, i.e. they became less hypometabolic, and it was not modified in the left hypothalamus (figure 2).

**Figure 3:**
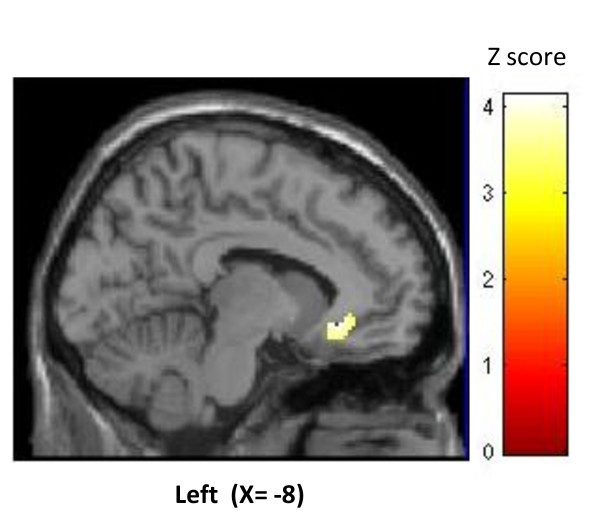
**Activation of perigenual cortex in ONS responders vs. non responders after 6 to 30 months stimulation (p < 0.05 FDR corrected)**. Result is displayed on a left sagittal section of a normalized MRI template.

When finally comparing responders and non-responders in the late phase, there was a significant hypermetabolism in previously identified perigenual ACC ipsilateral to the pain and stimulation side in responders (figure 3). No hypometabolic area differentiated responders from non-responders.

## Discussion

The therapeutic outcome after ONS in 1^st ^and 2^nd ^groups was overall similar, with good response as defined above in nearly 70% of patients [8,17]. A noticeable difference between the 2 groups was the latency to significant efficacy, which was on average shortened to 1 month in the 2^nd ^group compared to 3 months in the 1^st ^one. This remains compatible with a slow modulatory effect on the central nervous system. We ascribe faster efficacy in group 2 to a learning effect for investigators and to quicker optimisation of stimulator settings [8].

We will focus our discussion on the FDG-PET findings.

### PET findings - interpretation

#### Metabolic pattern in drCCH compared to HV

The enhanced FDG uptake found ipsilaterally in hypothalamus of drCCH patients respective to HV is in line with the reports showing increased activity in this area with H2^15^O-PET or fMRI during attacks [18-20]. However in our study all patients were scanned between attacks, i.e. at a time point when hypothalamic activation has not been reported yet. Another FDG-PET study of episodic CH comparing patients during and between bouts revealed no change in hypothalamic glucose uptake [21].

Areas belonging to the pain matrix like the cingulate gyrus or midbrain (periaqueductal grey - PAG) are classically activated in various pain states including headaches [5]. We also found activation in the cerebellum, in line with previous imaging studies showing consistent cerebellar activation across the spectrum of pain from visceral to somatic, acute to chronic [22]. Activation of cerebellar vermis and anterior lobe in drCCH may be particularly strong due to dense somatotopically arranged trigemino-cerebellar connexions [22], but also because there are direct connections between the ventro-posterior hypothalamic area and the cerebellum as shown by MRI tractography in a drCCH patient treated with hypothalamic DBS [23].

The perigenual ACC (PACC) is of particular interest. Contrary to our results, it was found hypometabolic compared to HV in episodic CH [21]. However, when the authors compared the same patients during and between bouts, the PACC was hypermetabolic during the bout despite the absence of an attack at the time of scanning.

Lower pons activation has been described during attacks of hemicrania continua, but not of CH [5]. In hemicrania continua, dorsal pontine activation is ipsilateral, like in our study, while posterior hypothalamic activation is observed on the opposite side, unlike in CH.

The pulvinar where we found ipsilateral activation in drCCH patients has not been a region of interest in CH before. Pulvinotomy and electrical stimulation of the pulvinar have been used successfully in the treatment of chronic pain in humans [24]. In functional neuroimaging studies, pulvinar hypermetabolism is either associated with pain state [25] or with pain relief after various procedures [24], including ONS in chronic migraine [13].

We have no straightforward explanation for the increased metabolism of the ipsilateral visual cortex in our patients. Such activation has not been reported hitherto. Photophobia ipsilateral to the pain is a frequent attack-associated symptom in various TACs, in particular CH [26]. Like in migraineurs, the visual cortex of CH patients might thus be more sensitive to light stimuli. This hypothesis seems unlikely, however, as all subjects were scanned in a dark room.

To summarize, the interictal FDG-PET hyperactive pattern of drCCH patients comprises areas reported to be hypermetabolic mainly during TAC attacks (ipsilateral hypothalamus and pons), but also during a bout of the disorder outside of an attack (perigenual ACC).

#### Short-term changes associated with ONS

We found no significant differences between PET recordings performed with stimulator ON or OFF within a 72 hour delay. This finding is similar to Matharu et al.'s [13] observations in chronic migraine, except that they were not able to scan the patients OFF and pain-free because of an almost immediate recurrence of pain after interrupting the stimulation. They concluded that central structures were not modulated in chronic migraine by ONS beyond the stimulation period.

Here, lack of short-term metabolic modification favours a slow neuromodulatory effect of ONS, as we suspected before [8]. Interestingly, in CCH patients treated with hypothalamic DBS, May et al. [27] found rapid metabolic changes with H_2_^15^O-PET in various brain structures involved in cluster headache and more generally in the pain matrix within only 10 minutes of switching the stimulator ON or OFF, but there were no clinical correlates suggesting that the therapeutic effect of DBS is also due to slow CNS changes.

Our subanalysis with a smaller significance level revealed a change in left lower brainstem metabolism. It might indicate a short term ONS effect in the trigemino-cervical complex. As expected from the neuro-anatomical connexions, the trigemino-cervical complex could relay stimulation to more rostral structures allowing for neuroplastic modulation of their activity. In comparison, a recent study of transcutaneous electrical nerve stimulation (TENS) in arthritic rats suggests that its antinociceptive effect is mediated by ascending activation of the opioid system originating in the ventrolateral PAG and projecting via the rostral ventro-medial medulla to the spinal cord [28].

#### Long-term changes associated with ONS

Hypermetabolism of most overactive areas in drCCH patients compared to HV decreased after ONS. This was particularly obvious for the anterior cingulate cortex, left pulvinar, left visual cortex, left lower pons, cerebellum and midbrain. Conversely, baseline hypometabolism of sensori-motor cortices increased after ONS. The noteworthy exception to these post-ONS metabolic changes is the ipsilateral hypothalamus. This is precisely the region activated during CH attacks on the side of the pain [18] and where increased gray matter density is found on voxel-based MRI between attacks [29]. Our findings in drCCH contrast with those by Sprenger et al [21] in episodic CH where no significant metabolic change was found in the hypothalamus, either outside or during the bout. If replicated, our data suggest that persistent hypothalamic activation is a hallmark of chronic CH. They may explain why attacks are non-remittent but also why some ONS-treated, pain-free patients still have autonomic attacks [8]. A similar conclusion was drawn for the dorsal rostral pons in chronic migraine following the finding of persistent activation in this area despite ONS-induced pain relief [13]. The persistence of an ipsilateral hypothalamic activation despite reduced attack frequency also confirms that ONS is no more than a symptomatic therapy, as already suggested by the recurrence of attacks after interruption of the stimulation [8].

The metabolic changes observed after ONS could be due to the stimulation itself or to the reduction of attack frequency. Our protocol did not allow to favour either mechanism. However, despite the small number of non-responders in our study, some insight might be gained from the comparison of patients who clearly responded to ONS and those who did not.

#### Perigenual ACC (PACC) activation in responders

Comparison of ONS responders and non-responders revealed increased FDG uptake in the PACC of the former. This area is of interest for several reasons. First, PACC plays a major role in central opioidergic pain control system. It is selectively activated during analgesia induced by the μ-receptor agonists fentanyl and remifentanyl compared with placebo [30], providing evidence that opioidergic analgesia is mediated by activation of descending antinociceptive pathways. Second, PACC was found hypometabolic respective to HV in episodic CH, but its activity increased significantly during the bout [21]. Knowing the pivotal role of PACC in descending pain control, these authors hypothesized that deficient endogenous antinociceptive mechanisms between bouts might predispose CH patients to the disorder and to its recurrence. Concordantly, Sprenger et al [31], using PET with the opioidergic ligand [11C]diprenorphine, demonstrated an inverse linear relationship between the duration of CH and opioid receptor availability in the rostral ACC (and ipsilateral hypothalamus). A recent case report by the same group of a drCCH patient in whom low dose levomethadone induced complete remission of attacks favours this hypothesis [32].

Compared to TENS that was shown to induce analgesia through activation of a PAG-RVM-spinal cord pathway [28], ONS could activate this descending pain-control pathway even further up-stream at the level of PACC. Its therapeutic effect in drCCH patients could thus be due to progressive restoration of activity in deficient opioidergic antinociceptive pathways.

#### Study limitations

We are well aware of some methodological flaws that may limit the strength of our findings.

First, the number of patients included is rather small. This is the case in most similar studies as drCCH patients are rare and ONS is an emerging treatment modality for which only 38 cases, including ours, have been published. In a much commoner condition like chronic migraine, metabolic imaging studies have been limited to less than 10 patients [13].

A second shortcoming is the dichotomy of the PET design. PET studies were not planned in our initial pilot study of ONS in 5 patients as the outcome was uncertain and the sample considered too small. As clinical efficacy was encouraging, we were allowed to recruit 6 additional patients, for whom imaging studies were planned prospectively. For greater sample size, patients from the 1^st ^group were also proposed to undergo PET. This explains why the latter had neither baseline nor 1 month scans and why long-term treatment periods vary between 6 and 30 months. We know since that there is no further clinical improvement after 6 months of ONS and that in most patients attacks recur after stimulation interruption whatever the duration of ONS [8]. This is why we decide to merge scans obtained between 6 and 30 months of ONS, albeit statistically questionable.

Finally, one may argue that the prophylactic drugs taken by the patients may have influenced the PET results. This cannot be ruled out, as HV did not take any medication. However, pharmacotherapy remained stable in all patients of the 2^nd ^group except one and was similar in ONS responders and non-responders.

## Conclusions

We confirm that ONS is effective and safe in drCCH, reducing attack frequency by ≥ 50% in more than 60% of patients, which is similar to the results obtained with hypothalamic DBS.

The FDG-PET results in our small sample appear consistent with the clinical impression that ONS exerts its beneficial effects via slow neuromodulatory processes in the central pain matrix. The finding of a possible selective perigenual ACC activation in responders compared to non-responders might advocate that ONS activates descending pain control systems in a top-down manner and restores an equilibrium in anti-nociceptive opioidergic pathways. We suggest for the first time that metabolic activity could be increased in ipsilateral posterior hypothalamus in chronic cluster headache patients outside of an attack. That ONS, as suspected on clinical grounds, does not cure drCCH, but merely acts as a symptomatic treatment is underlined by its inability to reduce this ipsilateral hypothalamic hyperactivity which is typically found during attacks in episodic cluster headache. Persistent hypothalamic activation might also explain why ONS-treated pain-free drCCH patients may still have autonomic attacks and why attacks rapidly recur after interruption of ONS.

## Abbreviations used in the text

18-FDG-PET: 18-Fluorodeoxyglucose-positron emission tomography; ACC: anterior cingular cortex; BA: Brodmann area; DBS: deep brain stimulation; drCCH: drug-resistant chronic cluster headache; HV: healthy volunteers; OFF: stimulator switched off; ON: stimulator switched on; ONS: occipital nerve stimulation; PACC: perigenual anterior cingular cortex; PAG: periaqueductal grey; TACs: trigeminal autonomic cephalalgias; TENS: transcutaneous electrical nerve stimulation

## Competing interests

The authors declare that they have no competing interests.

## Authors' contributions

All authors read and approved the final manuscript. DM participated to the clinical follow-up of patients, interpreted the PET results and drafted the manuscript. MAB analyzed the PET data and wrote the methodology. AF designed the PET study protocol. PYG participated to the clinical follow-up of patients. RH is head of the University Nuclear Medicine Department where patients underwent the PET scans. SL analyzed the PET data and did the matrix design. JS participated to the clinical follow-up of patients, interpreted the PET results and drafted the manuscript with DM.

## Author's information

DM, MD, is clinical chief associate at the University Neurology Department, Liège, Belgium.

MAB, MSc, is research fellow at the National Fund for Scientific Research (FNRS), Belgium.

AF, MD, PhD, is clinical chief at the University Neurology Department, Liège, Belgium.

PYG, MD, is research fellow at the University Neurology Department, Liège, Belgium.

RH, MD, PhD, is head of the University Nuclear Medicine Department, Liège, Belgium.

SL, MD, PhD, is senior research associate at the FNRS, Belgium.

JS, MD, PhD, is head of the Headache Research Unit at the University of Liège, Belgium and Professor of Neuroanatomy.

## Pre-publication history

The pre-publication history for this paper can be accessed here:

http://www.biomedcentral.com/1471-2377/11/25/prepub
